# Supplementary figures and images for: Evolution of interface binding strengths in simplified model of protein quaternary structure
Source: PLoS Comput Biol. 2019 Jun 3;15(6):e1006886. doi: 10.1371/journal.pcbi.1006886 (PMC6564041; doi:10.1371/journal.pcbi.1006886)

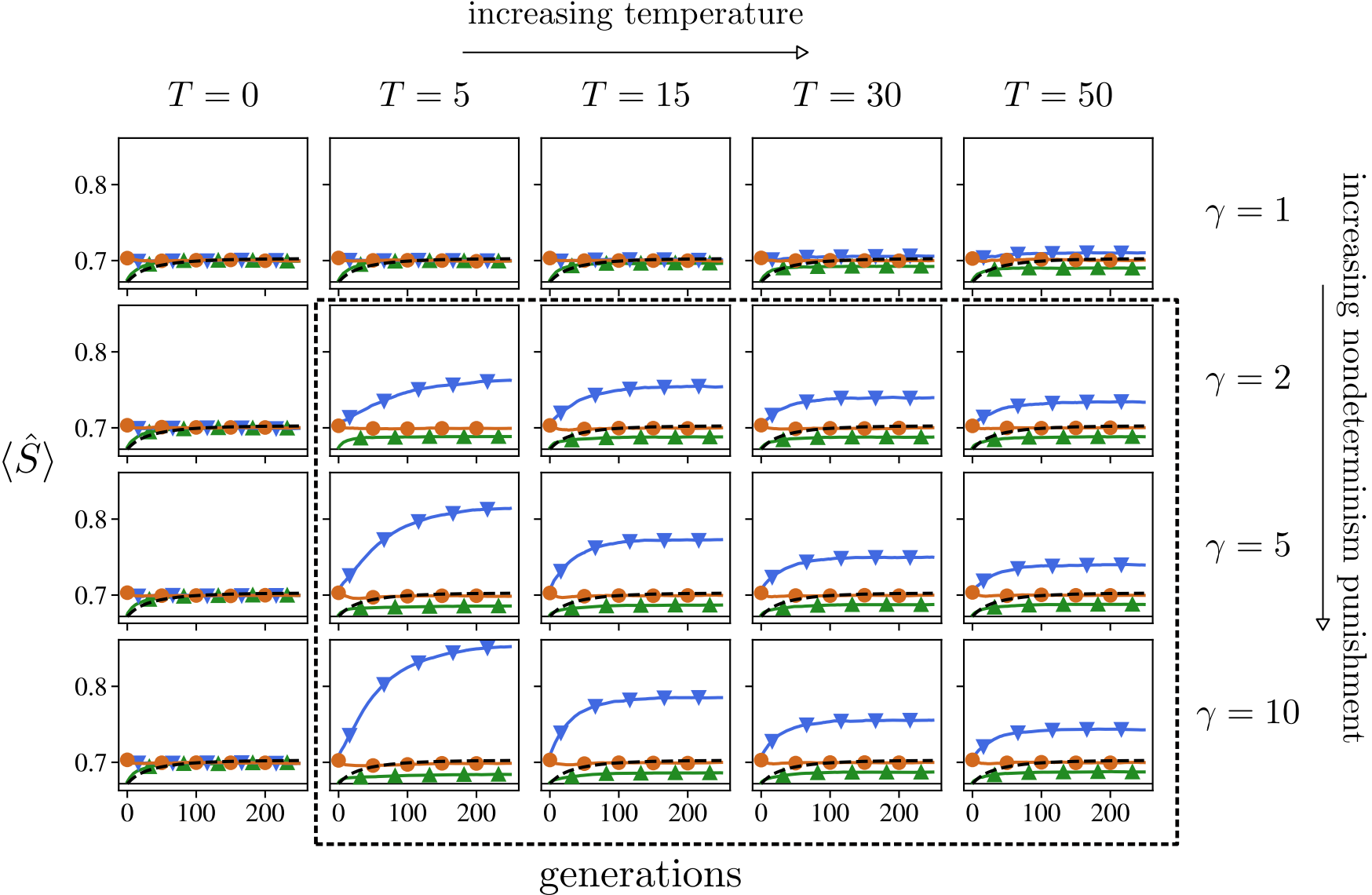

Supplement: S1 Fig — For all values of temperature T > 0 and nondeterminism punishment γ > 1 (in dashed box), where the parameter space enables stronger bonds to optimize determinism, the same qualitative observations hold as seen in the top right panel of Fig 4. The equilibrium values of interaction strength do depend on the selective pressure and temperature, but vary intuitively. (PDF) [file pcbi.1006886.s001.pdf]

12-mer

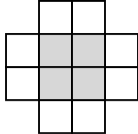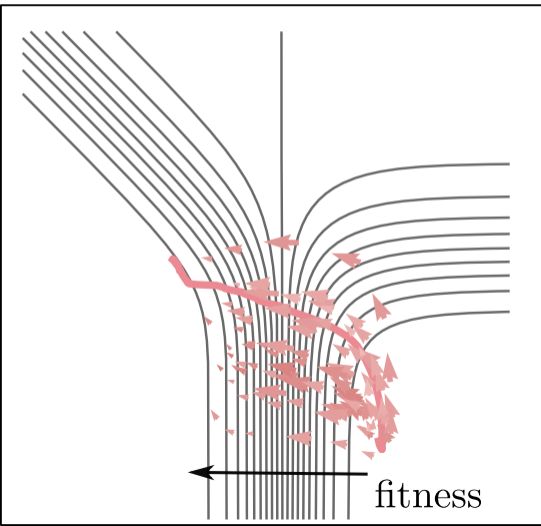

10-mer

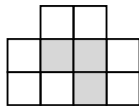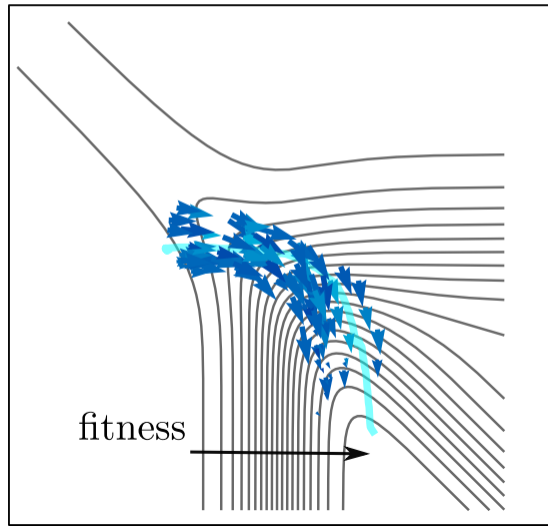

Supplement: S2 Fig — After switching the rewarded phenotype in the fitness landscape, average trajectories closely follow the determinism gradient of the relevant phenotype. Some trajectories switching from the 10-mer to the 12-mer (red) follow local gradients, increasing the C/B ratio first, as opposed to the more global optimum of lowering the A/C ratio. However, both paths tend to the same steady-state region of phase space. (PDF) [file pcbi.1006886.s002.pdf]
