# Supplementary material for: Evolution of interface binding strengths in simplified model of protein quaternary structure
Source: PLoS Comput Biol. 2019 Jun 3;15(6):e1006886. doi: 10.1371/journal.pcbi.1006886 (PMC6564041; doi:10.1371/journal.pcbi.1006886)
Supplement: S1 Text — (PDF) [file pcbi.1006886.s003.pdf]

Polyomino comparison

The assembly process forms structures of connected tiles called polyominoes. Subunit type and rotation may be important in identifying the phenotype, but global symmetries in the phenotype rarely match symmetries in the genotype. As such, it can be nontrivial to compare polyominoes assembled from different genotypes, or even repeated assemblies of the same genotype.

Polyomino encoding

A polyomino can be represented as a numeric string, encoding an unpadded bounding box starting from top left, moving row-wise, and terminating bottom right. Empty sites are denoted by 0. Occupied sites contain information on the placed subunit type T (counting from one) and rotation θ (counting how many $\pi/2$ clockwise rotations from the vertical) that were used during assembly. These sites have value $4T - 3 + \theta$, uniquely encoding type and rotation.

The polyomino encoding can be easily modified if different behaviour is desired. For example, if only phenotype shape was of interest rather than homogeneous or heterogeneous oligomers, the sites could be valued with 0 or 1 if empty or occupied respectively. In this way, the model can be adapted to what is considered "realistic" for the system under examination.

Minimal representation

In order to fairly compare polyominoes assembled by different genotypes, or even the repeated assemblies of the same genotype, polyomino representation has to be independent of genotypic ordering, absolute rotations, and reflections. The first step is to relabel the polyomino's numeric representation. The first occupied site is assigned $T = 1$ and $\theta = 0$, with this relabeling consistently propagated to all subunits of the same original type. Subunits originally labeled with $T = 1$ would be swapped with the original relabeled subunit type. The next occupied site that has not already been relabeled is assigned $T = 2$ and $\theta = 0$, likewise propagating the changes. This repeats until all occupied sites have been relabeled.

All unique rotations and reflections of a polyomino are then relabeled independently to determine the absolute minimum representation. As such, there can be up to 8 distinct representations, with the minimum representation having the lowest lexicographical ordering. Given two distinct representations $\underline{A}$ and $\underline{B}$ ($\underline{A} \neq \underline{B}$) of the same polyomino, $\underline{A}$ is more minimal than $\underline{B}$ if

$$\exists \underset{k}{\operatorname{argmin}}(A_k < B_k) \quad \text{S.T.} \quad (A_i \leq B_i) \forall i \in \mathbb{N}^{[1,k)}$$

An example is shown in Fig A, where two apparently different assembly graphs produce the same phenotype.

Alternative polyomino definitions

There are several levels of strictness for comparing polyomino structures, which can be straightforwardly incorporated. In addition to *free* polyominoes used in this work, there can also be *one-sided* (reflections distinct) or *fixed* (rotations and reflections distinct) polyominoes. For example, if using the fixed definition, then the minimal representation would be found purely from a single relabeling, while the one-sided definitions would require the 4 rotations to be compared. The lexicographical comparison is independent of these definitions, as it only alters the number of representations to compare. Likewise, the alternative encoding methods use the same definition for the lexicographical comparison.

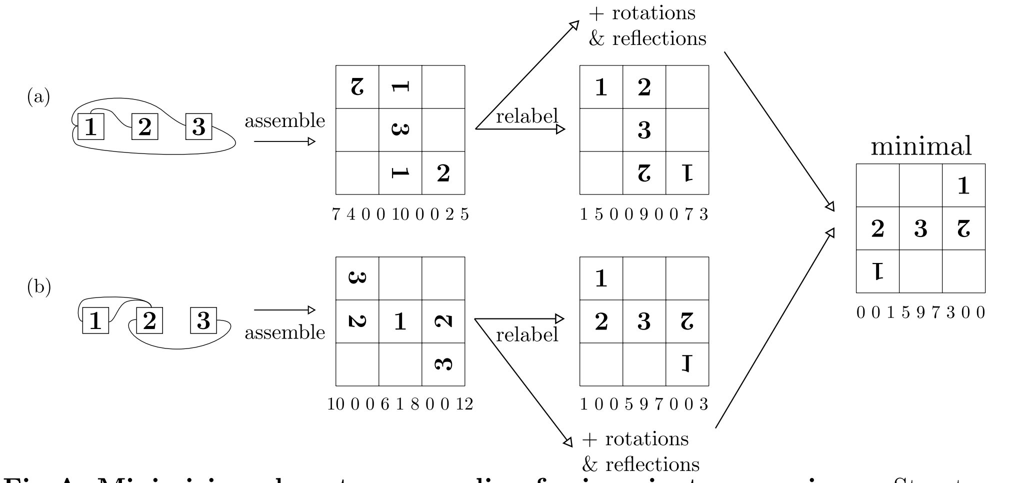

Fig A. Minimizing phenotype encoding for invariant comparisons. Structures can be represented with bounding boxes, with subunit type in bold numbers and rotated the appropriate amount. These are numerically encoded as described in text below the bounding box. For two genotypes represented with assembly graphs in (a) and (b), the assembled structures are shown. These are then relabeled, with other relabelings for rotations and reflections not shown explicitly. The minimal representation for each assembly graph is then found according to the lexicographical ordering, and is the same for both assembly graphs. As such, they have the same phenotype despite different assembly graphs.
