# Supplementary material for: Evolution of interface binding strengths in simplified model of protein quaternary structure
Source: PLoS Comput Biol. 2019 Jun 3;15(6):e1006886. doi: 10.1371/journal.pcbi.1006886 (PMC6564041; doi:10.1371/journal.pcbi.1006886)
Supplement: S2 Text — (PDF) [file pcbi.1006886.s004.pdf]

Markov evolution

Each state in the Markov process corresponds to a different interaction strength. The number of strength states k in the process is determined by $\hat{S}_c$ and L_I as

$$k = \lfloor (1 - \hat{S}_c) L_I \rfloor + 1$$

where $\lfloor . \rfloor$ is the floor function. Transition weights between these states depends on the likelihood of a random mutation strengthening (moving up a state) or weakening (moving down a state) an interaction. The probability of moving up a state from a random mutation decreases in stronger states, as $\hat{S}_i = 1 - \frac{k-i}{L_I}$. The fraction of the population in each state that mutates is given by $\mu \in (0, 1)$. This is visualized in Fig A.

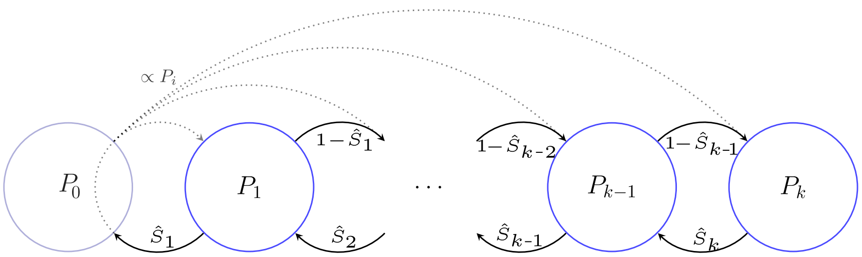

Fig A. Markov process on binding strength states. The fraction of the population in strength states P_i, with weighted transitions between them. Since any transition below the threshold state dies (P_0), that fraction of population is redistributed according to the fitness proportional selection. Since the contribution is proportional to P_i, these terms can be dropped without loss of generality.

The transition matrix $\mathbf{M}$ for the interaction strength states has a simple tridiagonal form and is given by

$$\begin{pmatrix} 1-\mu & \left(1-\hat{S}_1\right)\mu & & & \\ \hat{S}_2\mu & 1-\mu & \left(1-\hat{S}_2\right)\mu & & \\ & \hat{S}_3\mu & \ddots & \left(1-\hat{S}_{k-2}\right)\mu & \\ & & \hat{S}_{k-1}\mu & 1-\mu & \left(1-\hat{S}_{k-1}\right)\mu \\ & & & \hat{S}_k\mu & 1-\mu \end{pmatrix}$$

Strength state expectations

Although the transition matrix is not strictly positive, it is non-negative and forms a strongly connected directed graph (each state is reachable, eventually, from any state).

As such, the Perron-Frobenius theorem is satisfied. Crucially, this means an all-positive eigenvector exists, $\underline{p}_{PF}$, which gives the steady-state distribution of interaction strengths. The expectation of binding strength can then be found as

$$\langle \hat{S} \rangle = \underline{p}_{PF} \cdot \underline{\hat{S}} \quad \text{where} \quad \underline{\hat{S}} = [\hat{S}_1, \ldots, \hat{S}_K]$$

Transient expectations can also be calculated, assuming the population is initially localised entirely in the lowest strength state. After g generations, the expectation is

$$\langle \hat{S} \rangle_g = \mathbf{M}^g \underline{p}_0 \cdot \underline{\hat{S}} \quad \text{where} \quad \underline{p}_0 = [1, 0, 0, \ldots]^\intercal$$

The rate at which the transient expectations tend to the steady-state depends on μ (mixing time), and generally occurs on a much quicker time scale than the discovery of new interactions.
